# Optimized Cryopreservation of Mixed Microbial Communities for Conserved Functionality and Diversity

**DOI:** 10.1371/journal.pone.0099517

**Published:** 2014-06-17

**Authors:** Frederiek-Maarten Kerckhof, Emilie N. P. Courtens, Annelies Geirnaert, Sven Hoefman, Adrian Ho, Ramiro Vilchez-Vargas, Dietmar H. Pieper, Ruy Jauregui, Siegfried E. Vlaeminck, Tom Van de Wiele, Peter Vandamme, Kim Heylen, Nico Boon

**Affiliations:** 1 Laboratory of Microbial Ecology and Technology, Faculty of Bioscience Engineering, Ghent University, Gent, Belgium; 2 Laboratory of Microbiology, Faculty of Sciences, Ghent University, Gent, Belgium; 3 Microbial Interactions and Processes Research Group, Department of Medical Microbiology, Helmholtz Centre for Infection Research, Braunschweig, Germany; University of Missouri, United States of America

## Abstract

The use of mixed microbial communities (microbiomes) for biotechnological applications has steadily increased over the past decades. However, these microbiomes are not readily available from public culture collections, hampering their potential for widespread use. The main reason for this lack of availability is the lack of an effective cryopreservation protocol. Due to this critical need, we evaluated the functionality as well as the community structure of three different types of microbiomes before and after cryopreservation with two cryoprotective agents (CPA). Microbiomes were selected based upon relevance towards applications: (1) a methanotrophic co-culture (MOB), with potential for mitigation of greenhouse gas emissions, environmental pollutants removal and bioplastics production; (2) an oxygen limited autotrophic nitrification/denitrification (OLAND) biofilm, with enhanced economic and ecological benefits for wastewater treatment, and (3) fecal material from a human donor, with potential applications for fecal transplants and pre/probiotics research. After three months of cryopreservation at −80°C, we found that metabolic activity, in terms of the specific activity recovery of MOB, aerobic ammonium oxidizing bacteria (AerAOB) and anaerobic AOB (AnAOB, anammox) in the OLAND mixed culture, resumes sooner when one of our selected CPA [dimethyl sulfoxide (DMSO) and DMSO plus trehalose and tryptic soy broth (DMSO+TT)] was added. However, the activity of the fecal community was not influenced by the CPA addition, although the preservation of the community structure (as determined by 16S rRNA gene sequencing) was enhanced by addition of CPA. In summary, we have evaluated a cryopreservation protocol that succeeded in preserving both community structure and functionality of value-added microbiomes. This will allow individual laboratories and culture collections to boost the use of microbiomes in biotechnological applications.

## Introduction

In a bio-based economy, the exploitation of microbial resources represents a valuable solution for many of the current sustainability issues [1]–[6]. Both single strains and consortia of different microorganisms with various interconnected functions (i.e. microbiomes [7]) have been employed. The latter strategy has been gaining importance since the last decade [1], [3], [6], [8], [9] and in certain applications microbiomes are known to outperform pure cultures [9]–[13]. Hence, the use of mixed microbial communities is interesting both from a purely scientific point of view as well as from the viewpoint of practical applications. Nonetheless, no optimized approach to maintain a reproducible mixed community inoculum is available to date (even when correcting the inherent variability found in mixed bacterial communities (i.e. community dynamics [7]).

The majority of the existing protocols for long-term and stable storage have been described for axenic cultures, which is obviously linked with the almost exclusive focus of biological resource centers (BRC) on pure culture microorganisms [14], [15]. In non-BRC labs the preservation method of freezing at −80°C is preferred over freeze-drying or other drying techniques, because of the direct access to electrical freezers for most researchers and the straightforwardness of the procedure [16]. To avoid cellular damage during cryopreservation and subsequent thawing, a wide array of cryoprotective agents (CPA) has been applied. Of these, cryopreservation with dimethylsulfoxide (DMSO) is comparatively more successful than the commonly used glycerol [17]. Moreover, recent studies on preservation of fastidious pure cultures have shown the effectiveness of complex media for cryopreservation of methanotrophic bacteria (MOB) [18], aerobic and anaerobic ammonia-oxidizing bacteria (AerAOB and AnAOB) [19], [20] and nitrite oxidizing bacteria [21]. These complex cryopreservation media exploit the concerted protective effects of a fast penetrating CPA (DMSO) and the innate cryoprotective effects of carbon-rich media (Trehalose and Tryptic Soy Broth, TT). Apart from the choice of CPA, which has been indicated to be one of the most determining factors for cryopreservation success [17], a rigorous protocol for freezing, thawing, resuscitation and storage with as less temperature variations as possible is essential for successful cryopreservation [16].

To date, only a few methodologies for cryopreservation of non-axenic cultures have been described [20], [22]–[24]. Conversely, regardless of the increasing interest in processes driven by mixed microbial communities or microbiomes [3], [7], they are currently not readily available from any culture collection [14]. Among the described preservation methodologies, the use of DMSO and DMSO+TT as CPA has been evaluated for highly enriched anammox communities, without further evaluation of the community structure [20]. The preservation of the activity of anammox enrichments has also been evaluated at −60°C (not at −80°C) without evaluation of DMSO as a CPA but with evaluation of community changes by means of comparative FISH [25]. Cryopreservation of gel entrapped nitrifying sludge has also been evaluated [26] but not with DMSO as a CPA, nor with further evaluation of the community structure. The recovery of both activity and community structure (evaluated with DGGE) have been evaluated for cryopreserved denitrifying biomass [24]. However, DMSO nor TT were incorporated as CPA in the study design. Finally, cryopreservation of the oxygen-limited autotrophic nitrification/denitrification (OLAND) biofilm has been evaluated previously [22] but cryopreservation was not evaluated at −80°C nor with the use of DMSO or DMSO+TT as a CPA. To summarize, none of the described methodologies to this day have evaluated both the documented benefits of DMSO with or without carbon rich compounds as a CPA on both community composition and functionality.

This study presents the innovative implementation of a cryopreservation protocol, designed based on previous research [17]–[21], for stable storage of bacterial mixed cultures in order to retain both community composition and an associated key functionality over time. DMSO was chosen over glycerol as the CPA. The combination of DMSO and TT was evaluated as a separate CPA. Storage was performed at −80°C. Three different bacterial mixed communities were included: (i) a highly enriched co-culture of methane-oxidizing bacteria (MOB) and heterotrophs [27], (ii) a biofilm from the OLAND process [28] which contained both nitrifiers (aerobic ammonium-oxidizing bacteria, AerAOB and nitrite-oxidizing bacteria, NOB) and anoxic ammonium-oxidizing bacteria (or anammox bacteria: AnAOB) and (iii) a human fecal microbiome. These mixed bacterial cultures were selected based upon their relevance for science and industry. MOB mixed communities are the key drivers of a variety of biotechnological processes [29]: methane removal in gaseous or liquid wastestreams, production of added-value compounds from these wastestreams [2], [30], [31] or biodegradation of hazardous organic compounds [32]. The OLAND mixed communities form a one-stage sustainable nitrogen removal process removing ammonia from wastewaters with a low C/N ratio and ammonia loaded gas streams through a combination of partial nitritation and anammox [28], [33], [34]. Finally, the fecal microbiome opens perspective for pre- and probiotics testing and fecal transplantations [4]–[6].

## Materials and Methods

### 1. Biomass Sampling and Pretreatment

Prior to cryopreservation, biomass was harvested from three different sources with their own key specific functionality.

#### 1.1. MOB biomass

A methanotrophic co-culture was sub-cultivated from the original enrichment culture by Van Der Ha *et al.* [27] on NMS medium (with copper). Headspace air was replenished every three days, in a non-sterile fashion. Biomass was sampled from these communities growing in active methane oxidizing fed-batch reactors.

#### 1.2. OLAND biomass

OLAND is a one-stage autotrophic process removing ammonia from wastewaters with a low C/N ratio and ammonia loaded gas streams through a combination of partial nitritation and anammox [28], [33], [34].

The OLAND-biomass was harvested from a lab-scale rotating biological contactor (RBC) showing stable operation for several years [35]. The reactor is being operated at 34±1°C and has been fed with synthetic influent at a volumetric loading rate of 600 mg N L^−1 ^d^−1^ and a hydraulic residence time of 40 h. At the time of sampling, the average nitrogen removal efficiency was 77%. About 100 g of biofilm was harvested from the RBC discs by scraping. To remove all dissolved nitrogen compounds originating from the reactor liquid, the harvested biomass was washed with tap water in a sieve (pore size 250 µm).

#### 1.3. Fecal biomass

Following verbal consent, a stool sample of a healthy human volunteer was collected in a sealed, plastic container with an AnaeroGen bag to create an anaerobic environment. The sample was preserved within 2 h after defecation.

### 2. Experimental Setup

**Figure 1:**
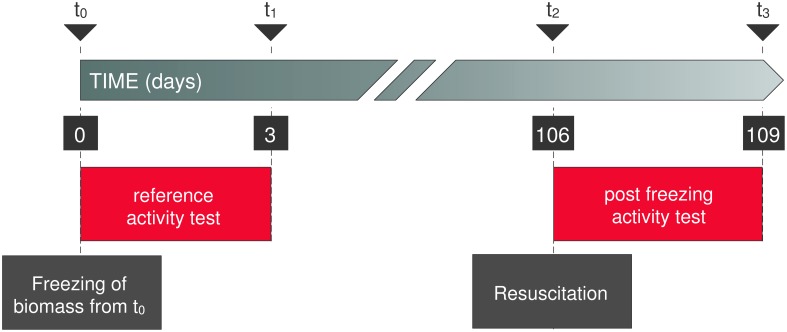
Timeline and sampling strategy of the cryopreservation setup. The time is shown in days. In red the activity tests are shown. The freezing and thawing are shown in dark grey. DNA sampling was executed at t_0_, t_1_ and t_3._

The experimental design, over a three month period of cryopreservation, is outlined in Figure 1. Each source of biomass at t_0_ was divided in three parts: one part was subjected to cryopreservation, with or without addition of CPA, another part was subjected to the reference activity test, and a final part was sampled for DNA extraction and biomass quantification. At the end of the reference activity test, biomass was again sampled for DNA extraction and biomass quantification (t_1_). After 106 days, biomass was resuscitated (t_2_) and used as inoculum for the post-freezing activity test. At the end of the post-freezing activity test biomass was sampled again for quantification and DNA extraction (t_3_). DNA sampling at this point allows to investigate the active community after resuscitation and a standard batch activity test.

#### 2.1. Storage conditions

Prior to cryopreservation and activity testing, all mixed cultures were cultivated in their appropriate growth media (described in section 2.3). Cultures were harvested from running (fed-) batch reactors or human fecal matter. Biomass was transported in under one h to the cryopreservation location on coldpacks (approx. 4°C). Biomass was stored in quadruplicate 50 mL falcon tubes for each cryopreservation condition (see Table 1), to allow for an adequate amount of inoculum to be preserved to execute activity measurements and reactor startup immediately after resuscitation [20].

**Table 1:** Cryoprotective agents (CPA) used in the crypreservation design.

Treatment designation	CPA content
No CPA	Distilled autoclaved tap water
DMSO	Distilled autoclaved tap water and DMSO to a final concentration of 5% (v/v) DMSO
DMSO + TT	TT medium (1% (w/v) trehalose, 0.3% (w/v) tryptic soy broth (TSB)) and DMSO to a final concentration of 5% (v/v) DMSO

In each falcon tube either 20 mL of liquid broth for the MOB biomass (previously cultivated on NMS) or 8 g of wet weight for the OLAND and fecal biomass was added. No particular precautions were made to avoid exposure of the anaerobized fecal slurry and OLAND biofilm to air in preparation of the 50 mL falcon tubes for freezing [20]. To the OLAND and fecal biomass, 20 mL of autoclaved tap water (Ghent, Belgium) or basal medium [36] was added, respectively. Immediately upon arrival at the cryopreservation site, 20 mL of the selected CPA (Table 1), was added and gently mixed. Addition of CPA was performed at 4°C to decrease DMSO toxicity. Biomass was allowed to equilibrate with the added CPA for 30 minutes at room temperature (21°C). Immediately after, biomass was transferred to −80°C. The falcon tubes were stored in non-insulated cryopreservation boxes in an aluminum rack placed in the −80°C ULT freezer. The freezing and thawing rates in medium with 5% DMSO at −80°C were similar to values determined by the authors in a previous experiment [19] as an identical protocol and equipment were used. In this experiment it was shown that the rates of freezing to −80°C were much lower than in liquid nitrogen, while thawing rates were similar. A slower freezing rate is beneficial for preservation success, as a rapid cooling can increase the chance of intracellular ice formation, leading to cell death.

#### 2.2. Resuscitation conditions

The last step in every preservation protocol is the resuscitation of preserved biomass so that cells again become active and are able to reproduce [16]. Samples were thawed in a warm water bath at 37°C. Because of cytotoxicity of DMSO, the samples were removed from the warm water bath immediately upon thawing for centrifugation at 4°C at 7000 g for 15 minutes after which the supernatant was discarded. The pellet was then resuspended in fresh medium, and 50% (v/v) TT medium was added to the corresponding vials. After resuspending, cultures were incubated for one h at room temperature. Then the samples were centrifuged as described above following pellet resuspension in 20 mL of their respective media.

### 3. Activity Screening Setup

#### 3.1. MOB biomass

A total liquid volume of 200 mL was used to have sufficient amounts of methane and oxygen in the headspace for a 96 h incubation in 1.15 L bottles. At the start of each incubation, 20% (v/v) of methane (99.95% pure, Air Liquide, Liège, Belgium) was added to the headspace of the bottles. All cryoprotective conditions were incubated in duplicate on both NMS medium (1 g L^−1 ^MgSO_4_·7H_2_O, 1 g L^−1^ KNO_3_, 0.15 g L^−1^ CaCl_2_·2H_2_O, 0.005 g L^−1^ FeNaEDTA, 1.43 g L^−1^ Na_2_HPO_4_·12H_2_O, 0.55 g L^−1^ KH_2_PO_4_ and 0.1% (v/v) of trace elements solution) with copper and dNMS medium (0.4 g L^−1 ^MgSO_4_·7H_2_O, 0.4 g L^−1^ KNO_3_, 0.06 g L^−1^ CaCl_2_·2H_2_O and 2.87 g L^−1^ Na_2_HPO_4_·12H_2_O, 1.1 g L^−1^ KH_2_PO4. Other constituents are given for NMS). The trace elements solution is described in Table S1, and contains 2.5 g L^−1^ CuSO_4_·5H_2_O, among other nutrients. The bottles were placed on a shaker (120 rpm) for 48 h with daily sampling for cell dry weight (10 mL) and headspace gas (2 mL).

Specific methane oxidation rate (MOR, mmol CH_4 _g^−1^ VS d^−1^) was determined as the amount of methane consumed divided by the average volatile solids (VS) concentration over the activity test of 48 h, and was the key activity for MOB mixed culture.

#### 3.2. OLAND biomass

The aerobic batch experiments for AerAOB and NOB activity were performed in 250 mL erlenmeyer flasks with 100 mL working volume, where 0.1 g L^−1^ of nitrogen added as NH_4_Cl and a buffering solution (1 g L^−1^ NaHCO3, 3.4 g L^−1^ KH_2_PO_4_ and 4.4 g L^−1^ K_2_HPO_4_) were supplied to the biomass (∼0.2 g VSS per erlenmeyer). The flasks were incubated on a shaker at 34°C while pH and dissolved oxygen concentration were monitored: samples for ammonium, nitrite and nitrate analyses were taken each 4 h. For the anoxic batch tests (AnAOB activity), 120 mL serum flasks were used, containing 80 mL of mixed liquor. Once the biomass (∼0.2 g VSS per flask) and a buffering solution (final concentrations 1 g NaHCO_3_ and 0.04 g L^−1^ KH_2_PO_4_) were added, the flasks were closed with rubber stoppers and flushed with N_2_ gas (30 cycles of 800 mbar overpressure, 900 mbar underpressure). Then, flushed substrate solutions containing NH_4_Cl and NaNO_2_ were added (final concentrations 0.1 g L^−1^ NH4-N and 0.1 g L^−1^ NO_2_-N). Further incubation and sampling were performed as described for the aerobic batch experiments. Due to a missing sampling point (t_1_) and the pooling of the biomass, statistical analyses were not completed for the community data acquired.

#### 3.3. Fecal biomass

The fresh or preserved (after removal of cryoprotectant medium) fecal sample was diluted (20%, w/v) and homogenized with sterilized phosphate buffer (0.1 M, pH 7.0), containing 1 g L^−1^ sodium thioglycolate as the reducing agent. The particulate material was removed by centrifugation (2 min, 500 g) and the supernatant of this pretreatment was used as an inoculum for the batch tests.

Experiments were performed in 120 mL serum flasks flushed with N_2_ (as described for the anoxic OLAND activity tests) with basal medium [36]. Forty mL of basal medium and 10 mL of the fecal inoculum were incubated at 37°C under continuous shaking (120 rpm) for 36 h. The activity parameter that was evaluated for the fecal community was short-chain fatty acid (SCFA) production. The total SCFA concentration was calculated as the sum of concentrations of acetate, propionate, butyrate, isobutyrate, valerate, isovalerate, caproate and isocaproate.

### 4. Physicochemical Analyses

#### 4.1. Biomass quantification

Specific activity, biomass concentration was assessed as cell dry weight based on a determination of total (suspended) solids (T(S)S) and volatile suspended solids (V(S)S) according to Greenberg *et al.* [37].

#### 4.2. Headspace gas composition

At the startup and before every liquid sampling event, gas samples were taken and gas pressure was measured using a tensiometer (Infield 7 with T1Kc sensor head, UMS, München, Germany). Two mL of headspace sample was transferred to and injected on a Compact GC (Global Analyzer Solutions, Breda, The Netherlands) equipped with one channel having a thermal conductivity detector (TCD) following a Porabond pre-column attached to a Molsieve SA column. This allowed for accurate determination of the concentration of O_2_, N_2_, CH_4_ and CO_2_ in the headspace of the cultivation reactors [2].

#### 4.3. Ammonium, nitrite, nitrate and SCFA analyses

Dissolved ammonium concentrations were determined by a direct colorimetrical method with the Nessler reagent at 425 nm [37]. Nitrate and nitrite concentrations were analyzed using an ion chromatograph (IC 761 Compact IC, Metrohm, Herisau, Switzerland) equipped with an electrochemical conductivity detector following a Metrosep A Supp 5–150 column (Metrohm, Herisau, Switzerland) and a Metrosep A Supp 4/5 guard column (Metrohm, Herisau, Switzerland). The mobile phase was 3.2 mM Na_2_CO_3_, 1.0 mM NaHCO_3_ and 5 volume percent acetone at a flow rate of 0.7 mL min^−1^. Volatile fatty acids were analyzed as described previously [38].

### 5. Microbial Identification and Quantification

To identify the mixed bacterial community constituents, Illumina 16S rRNA gene sequencing was performed. DNA was extracted using the FastDNA SPIN kit for soil (MP Biomedicals, Brussels, Belgium) according to the instructions of the manufacturer for the MOB and OLAND samples and by using the CTAB method [39] for the human fecal material samples.

The preparative amplification, gel purification and equimolar pooling for Illumina amplicon sequencing was performed as described before [40]. Unidirectional Illumina amplicon sequencing was executed with 16S rRNA gene primers for the V5–V6 hypervariable regions as described before [41].

A total of 544568, 83754 and 170617 sequence reads were obtained for the 20 MOB samples, 4 OLAND samples and 9 fecal microbiome samples, respectively. A quality filter program that runs a sliding window of 10% of the read length over the read and calculates the local average score based on the Phred quality scores of the fastq file, trimmed 3′-ends of the reads that fall below a quality score of 15 (http://bioinformatics.ucdavis.edu/index.php/Trim.pl). Only reads of a minimum of 149 nt in length (29 nt of primer and barcode sequence and 120 nt of 16S rRNA gene sequence) were further analyzed. All truncated reads that had an N character in their sequence, any mismatches within primers and barcodes or more than 10 homopolymer stretches were discarded. All sequences from each sample present in the different libraries were split into different files according to their unique barcode.

A representative read was further considered if a) it was present in at least one sample in a relative abundance >1% of the total sequences of that sample or b) was present in at least 2% of samples at a relative abundance >0.1% or c) present in at least 5% of samples. Phylotype representatives were then generated by clustering at 98% similarity (1 mismatch) using the mothur pre.cluster program [42]. This reduced the number of representative reads to a computational manageable level without curtailing the fine scale community composition [40]. The final sequences used for this study are available as supplemental material (Datasets S1 through S3).

Additionally, for the MOB mixed community, a diagnostic microarray analysis targeting the *pmoA* gene (a gene encoding for the methane monooxygenase, a key enzyme in methane oxidation) was performed as described in detail previously [43]–[45]. The probe identification was given previously [45].

### 6. Data Processing and Visualization

All statistical data analyses and graphing of community structure were performed with the statistical software R, version 3.0.2. for Windows (http://www.r-project.org) [46]. Multiple comparisons were executed using the Kruskal-Wallis rank sum test from the R base package stats. If the null hypothesis of equality of location parameters of each group distribution was rejected, nonparametric relative contrast effects [47] were estimated to assess significant differences between groups with Tukey contrasts, unless stated otherwise. Graphing of the functionality data was performed using SigmaPlot for Windows version 12.0 (Systat software, Inc.).

Phylogenetic trees were constructed after sequence alignment using mothur, version 1.31.2 [42]. Alignments were made using the align. seqs command with the reference Silva alignment provided on the mothur website (http://www.mothur.org/wiki/Silva_reference_files). RAxML [48] was used to construct a majority rule bootstrap consensus tree with the GTR+GAMMA substitution model. One thousand bootstrap iterations were executed using the parallelization offered in the Pthreads-based version of RAxML [49]. The Newick-formatted output tree was subsequently loaded into iTol (http://itol.embl.de) for data visualization [50]. Classification of sequences was executed with the mothur implementation of the naïve Bayesian classifier [51] with a threshold of 0.65 with either RDP release 9 [52] reference taxonomy or the Greengenes reference taxonomy suggested by Werner and colleagues [53], without trimming to the sequencing region. Unless stated otherwise, the sequence count data were randomly subsampled to the sequence count of the sample with the lowest sequence count for each of the mixed communities separately. Rarefaction curves show that at this cutoff all samples were sequenced deep enough to cover biodiversity (Figure S8).

When means are reported, they are always reported as mean ± standard deviation (µ±σ) and the number of replicates (*n*) is given between brackets. Multiple comparisons were made using nonparametric relative contrast effects [54] with Tukey contrasts and a logit asymptotic approximation as implemented in the R package nparcomp (http://cran.r-project.org/web/packages/nparcomp/index.html), unless otherwise stated.

## Results

For each of the three mixed communities the impact of the addition of a CPA during cryopreservation is outlined below. First specific activity recovery of a key activity of the mixed culture is given, followed by the results of the recovery of OTUs directly contributing to the chosen key activity (functional community members) after which the recovery of every single OTU, regardless of classification as a functional community member, is evaluated for their presence or absence at each time point and condition of the experimental design (Figure 1). Finally the changes in community structure are evaluated using the abundance-based Jaccard index.

### 1. Methanotrophic Community (MOB)

**Figure 2:**
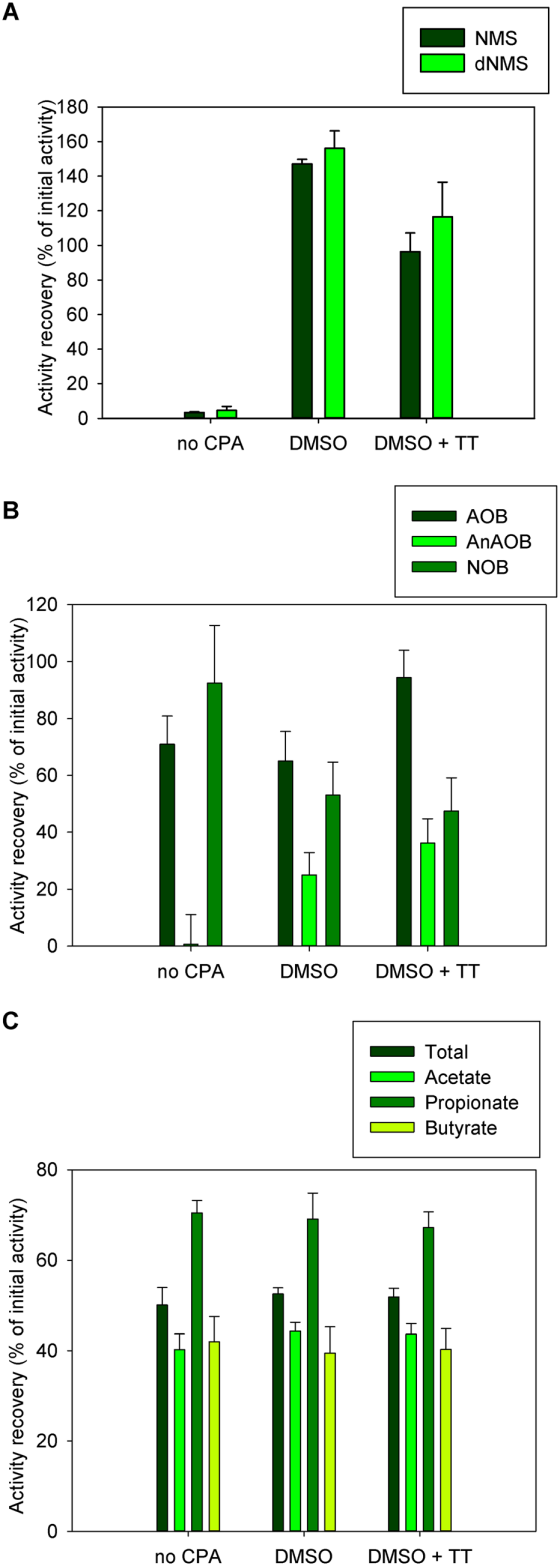
Functionality recovery after cryopreservation. Error bars represent error-propagated standard errors A) the MOB community on NMS and dNMS cultivation medium. The activity recovery was the percentage of specific MOR (mmol CH_4 _g^−1^ VS d^−1^) from the pre-freezing activity test (t_0_ to t_1_) that was obtained in each experimental condition in the post-freezing activity test (t_2_ to t_3_) B) The different functional members in the OLAND community. The activity recovery was the percentage of specific activity (mg N g^−1^ VSS d^−1^) for either aerobic or anaerobic ammonium oxidation (AOB and AnAOB) or nitrite oxidation (NOB), from the pre-freezing activity test (t_0_ to t_1_) that was obtained in each experimental condition in the post-freezing activity test (t_2_ to t_3_) C) short chain fatty acid production by the fecal microbiome. The activity recovery was the percentage of SCFA produced in the pre-freezing activity test (t_0_ to t_1_) that was obtained in each experimental condition in the post-freezing activity test (t_2_ to t_3_). Total SCFA, acetate, propionate and butyrate were measured.

Two cultivation media (NMS and dNMS) were evaluated with the MOB mixed culture. The key specific activity (MOR) was comparable to the original activity, when a CPA was added prior to cryopreservation (Figure 2A), on both media. When no CPA was added, the average specific activity over 48 h was significantly lower than the original activity on both media (p<0.0001). The largest activity recovery was obtained when only DMSO was added as a CPA both with NMS (147.2±2.6%) and dNMS (156.1±10.1%), which exceeded the original activity. When DMSO+TT was used as a CPA, the original specific activity was obtained on NMS (96.4±10.7%) and dNMS (116.5±20%). Both on NMS and dNMS with DMSO+TT as a CPA the specific activity was not significantly different from the initial activity (p = 1).

All OTUs classified as methanotrophic *Proteobacteria* (57 out of 117 OTUs) were classified into two MOB families: either *Methylococcaceae* (*Gammaproteobacteria* or type I MOB) or *Methylocystaceae* (*Alphaproteobacteria* or type II MOB). Also one OTU was classified as a non-proteobacterial MOB, part of the *Verrucomicrobia* phylum, namely *Candidatus* Methylacidiphilum. While the *Methylococcaceae* family was an abundant community constituent (up to 46% of all sequences of the inoculum), the *Methylocystaceae* and *Candidatus* Methylacidiphilum were less abundant (Figure S4, Figure S7). All of these methanotrophic taxa were detected in the mixed culture before and after cryopreservation, irrespective of the added CPA (Figure S4). However, the relative abundances of the MOB OTUs among all experimental conditions simultaneously (t_0_, t_1_ and t_3_) were significantly different for each MOB family (p<0.0001). More specifically, the relative abundance of *Methylococcaceae* within dNMS pre (t_1_) and dNMS DMSO (t_3_) did not significantly differ from the inoculum (t_0_, p = 1). There was also no significant difference in *Methylococcaceae* relative abundance between the dNMS pre (t_1_) and dNMS samples at t_3_ with DMSO and DMSO+TT (p = 1). However, on NMS, all conditions at t_3_ differed significantly from t_0_ and t_1_, regardless of CPA addition (p<0.0001). There were no significant differences in relative abundance of *Methylocystaceae* between t_3_ and the initial inoculum (t_0_, p = 1), although the NMS samples showed a significant difference between t_3_ and t_1_ in samples with CPA addition (p<0.0001). No significant differences (p = 1) were found in the relative abundance of *Candidatus Methylacidiphilum* between t_0_ on t_3_ only when DMSO was used as a CPA and incubation was performed on NMS. Between t_1_ and t_3_ on NMS no significant differences were found when DMSO+TT or no CPA was used (p = 1). On dNMS, DMSO+TT maintains the relative abundance between t_1_ and t_3_ (p = 1).

**Figure 3:**
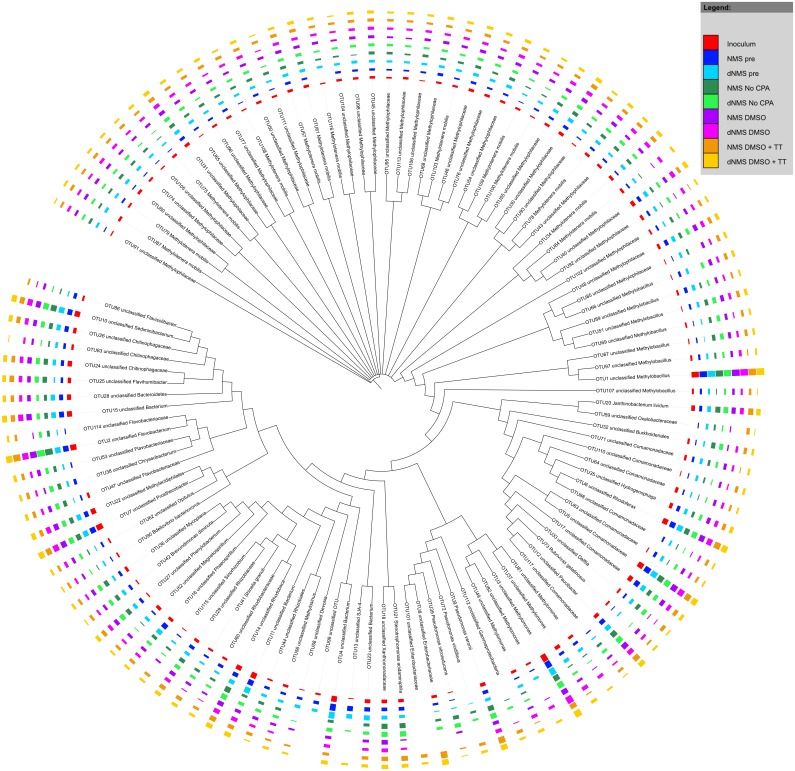
Phylogenetic tree of OTU consensus sequences in the MOB samples. Sequences were aligned using the mothur implementation of the NAST algorithm with the Silva v102 reference alignment. RAxML was used to construct an extended majority rule bootstrap consensus tree with the GTR + GAMMA substitution model and 1000 bootstrap iterations. This bootstrap consensus tree was visualized using iTol. The colored bars represent treatment-wise means (n = 2 except for inoculum n = 1 and NMS pre n = 3) of the log transformed absolute abundances with the log transformation as suggested by Anderson and colleagues [74] with base 10. Before transformation the samples were rarefied to the lowest sequence count after removal of anomalous sample NMS1. Red arrows indicate OTUs classified as methanotrophic bacteria. Black arrows point out OTUs that were not detected in specific experimental conditions. Classification was done based upon the Greengenes taxonomy (adapted to mothur from [53]) with the naïve Bayesian classifier implemented in mothur (Wang algorithm).

The main constituents of the mixed MOB culture were from the *Methylophilaceae*, *Flavobacteriaceae*, *Methylococcaceae*, *Comamonadaceae*, *Verrucomicrobiaceae*, *Chitinophagaceae* and *Enterobacteriaceae* families (Figure S1). Differences in relative abundance of individual community members were apparent however; only 21% of OTUs (representing only 2.5% of total sequence counts) were not detected in at least one of the experimental conditions (Figure 3).

**Figure 6:**
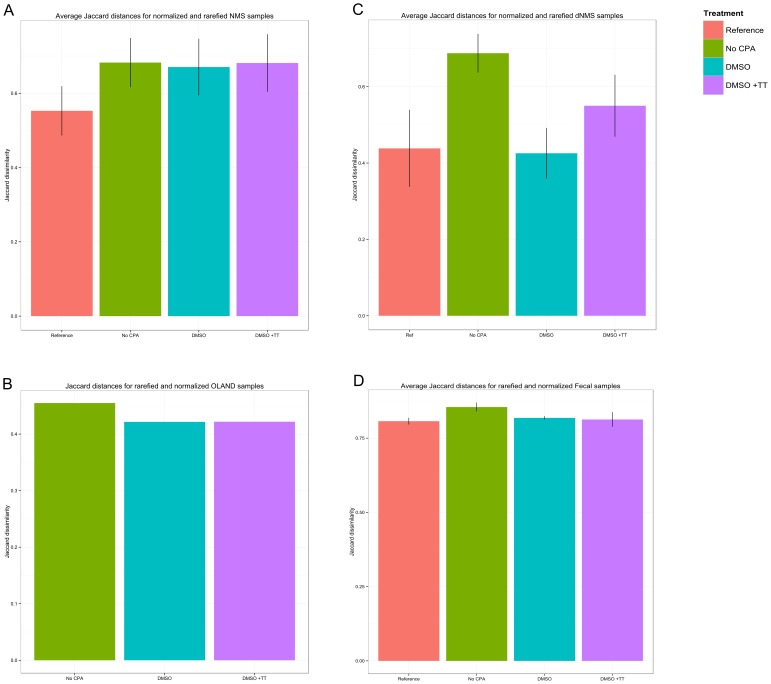
Average abundance-based Jaccard (Ružička) distances between experimental stages and conditions. The distances are displayed for MOB biomass cultivated on NMS (A), dNMS (C), OLAND biomass (B) and fecal biomass (D). The reference represents the distance between t_0_ and t_1_ whilst the other bars represent the distance between t_0_ and the several conditions at t_3_. Error bars (for MOB and fecal samples) represent standard deviations (n = 4 in A, n = 6 for reference in C and 4 for the other means, n = 4 in D). No reference is available for the OLAND biomass because time point t_1_ was not assessed using Illumina.

Cryopreservation was associated with greater community dissimilarities on NMS, regardless of the addition of a CPA (Figure 6A). Nonetheless, these dissimilarities (t_0_–t_3_) were not significantly different from the community dissimilarity of the first activity test (t_0_–t_1_, p = 0.12). With dNMS, the differences were significant (p = 0.01). More specifically, the dissimilarity with the inoculum was lower when a CPA was added. This dissimilarity was within the range of the dissimilarity of the pre-freezing activity test (t_0_–t_1_) when DMSO was used as a CPA (Figure 6C). The community dissimilarities in the reference (t_0_–t_1_), DMSO (t_1_–t_3_) and DMSO+TT (t_1_–t_3_) samples were not significantly different (p_ref-DMSO_ = 0.99, p_ref-DMSO+TT_ = 0.50, p_DMSO-DMSO+TT_ = 0.57) while the dissimilarities between the reference and the samples with no CPA did significantly differ (p<0.0001).

Overall, partial constrained correspondence analysis (pCCA) showed that the global community structure of samples at t_3_ with an added CPA is closer to the inoculum (t_0_) and results after the first incubation (t_1_) and significantly (p = 0.01) correlates with an increased recovery of MOR (Figure S9). This analysis ‘partials’ out the effect of the medium and allows to observe only the effect of CPA addition.

### 2. Oland Mixed Community

The functional autotrophic microorganisms in OLAND include AerAOB and AnAOB (Figure 2B). AerAOB had an complete specific activity recovery when DMSO+TT was used as a CPA (94.4±9.6%, p = 1) whilst only 71.0±9.9% was obtained without addition of CPA and only 65.1±10.4% was obtained when DMSO without TT was used as a CPA. AnAOB activity was recovered up to 36.1±8.6% with DMSO+TT as a CPA and up to 25.0±7.7% with only DMSO as a CPA. The initial specific activity was not recovered without CPA addition (0.7±10.4%). Finally NOB activity was best retained when no CPA was added (92.4±20.3%, p = 0.95) whilst only 53.1±11.6% or 47.4±11.7% was recovered with DMSO and DMSO+TT, respectively.

All OTUs that could be classified as AerAOB were representatives of the *Nitrosomonadaceae* family, more specifically *Nitrosomonas* sp. The AnAOB-OTUs were represented by the *Brocadiaceae* family, and more specifically by *Candidatus* Brocadia sp. NOB were representatives of *Nitrospiraceae* family, more specifically *Nitrospira* sp. Overall, the conditions where DMSO+TT was added as a CPA allowed for the best recovery after cryopreservation of all of the OLAND “functional” partners. The differences in relative abundance between no CPA and DMSO were rather minute (Figure S5).

**Figure 4:**
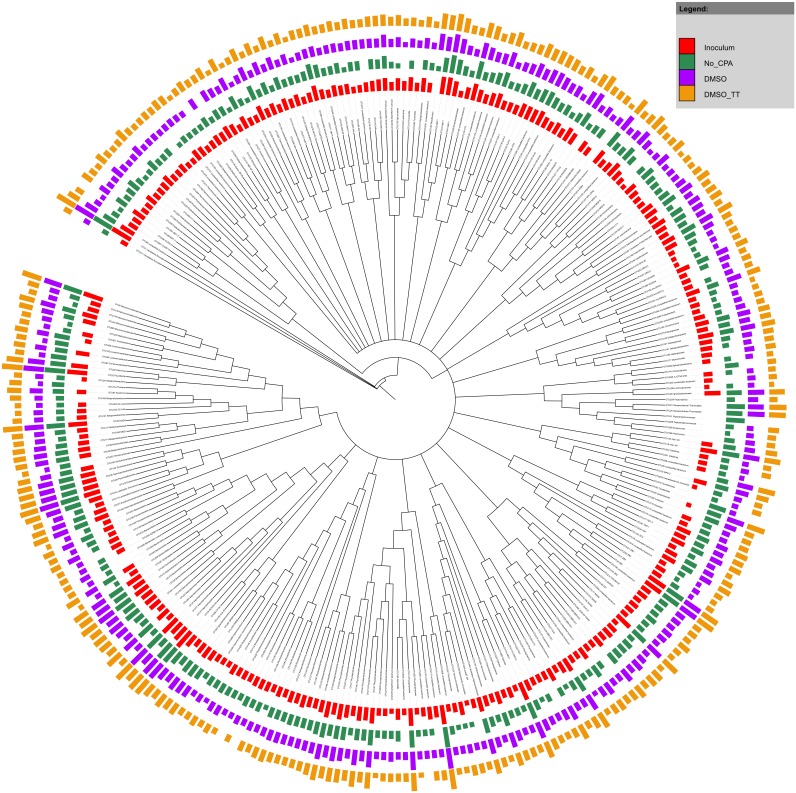
Phylogenetic tree of OTU consensus sequences in the OLAND samples. Sequences were aligned using the mothur implementation of the NAST algorithm with the Silva v102 reference alignment. RAxML was used to construct an extended majority rule bootstrap consensus tree with the GTR + GAMMA substitution model and 1000 bootstrap iterations. This bootstrap consensus tree was visualized using iTol. The colored bars represent log transformed absolute abundances with the log transformation as suggested by Anderson and colleagues [74] with base 10. Before transformation the samples were rarefied to the lowest sequence count. Black arrows point out OTUs that have a differential presence among experimental conditions. Classification was done based upon the Greengenes taxonomy (adapted to mothur from [53]) with the naïve Bayesian classifier implemented in mothur (Wang algorithm).

The main constituents of the OLAND mixed community were representatives of the *Comamonadaceae*, *Flavobacteriaceae*, *Nitrosomonadaceae*, *Rhodocylaceae* and *OD1 incertae sedis* families as well as the *Bacteroidetes* order (Figure S2). Overall, most OTUs occurred in each condition irrespective of CPA addition, and only 15% of OTUs (representing 5.3% of total sequences) were not detected in at least one of the experimental conditions (Figure 4). Most of the OTUs that did not occur in every condition required the addition of a CPA to persist after cryopreservation. In the samples where a CPA was added, global community dissimilarity to the inoculum was lower, regardless of the type of CPA used (Figure 6B).

### 3. Fecal Microbiome

The overall SCFA concentration profile remained nearly identical (Permutation Hotelling T^2^ p-value: 0.69) between t_1_ and t_3_ (Figure 2C), although the total concentration was significantly lower (p<0.0001). Initial metabolic activity was reduced to 50.1±3.9% if no CPA was employed, and to 52.5±1.4% with DMSO or to at 51.9±1.9% with DMSO+TT (p = 0.58). This decrease in total SCFA levels primarily originated from a decrease in acetate from 25.4±0.7 mM (n = 2) to 10.2±0.7 mM (n = 4) without CPA (p<0.0001), 11.2±0.6 mM (n = 3) on DMSO (p<0.0001) and 11.1±0.8 mM (n = 4) on DMSO+TT (p<0.0001). Compared to the initial levels (4.98±0.01 mM (n = 2)) propionate levels were highest without CPA addition (70.5±2.8%, 3.5±0.1 mM (n = 4)) and only slightly lower with DMSO (69.1±5.7%, 3.4±0.3 mM (n = 3), p = 0.51) or DMSO+TT (67.3±3.5%, 3.4±0.2 mM (n = 4), p = 0.87). Finally, the concentration of butyrate was highest with DMSO+TT (1.8±0.2 mM (n = 4)), which was 42.0±5.5% of the initial 4.2±0.07 mM (n = 2) while DMSO and no CPA resulted in 1.7±0.2 mM (n = 3) and 1.7±0.2 mM (n = 4), respectively.

**Figure 5:**
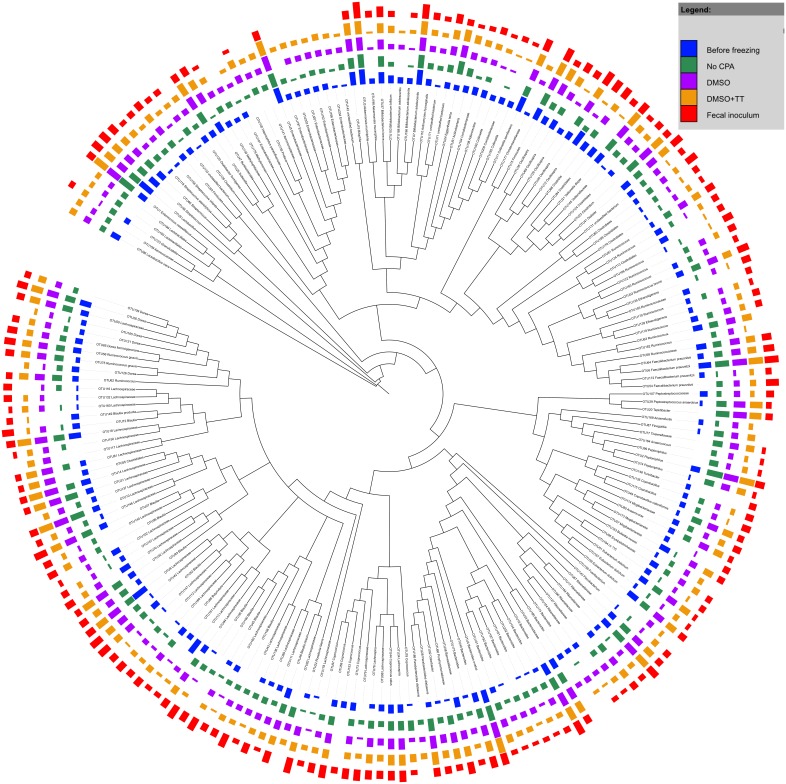
Phylogenetic tree of OTU consensus sequences in the fecal material samples. Sequences were aligned using the mothur implementation of the NAST algorithm with the Silva v102 reference alignment. RAxML was used to construct an extended majority rule bootstrap consensus tree with the GTR + GAMMA substitution model and 1000 bootstrap iterations. This bootstrap consensus tree was visualized using iTol. The colored bars represent treatment-wise means (n = 2 except for fecal inoculum n = 1) of the log transformed absolute abundances with the log transformation as suggested by Anderson and colleagues [74] with base 10. Before transformation the samples were rarefied to the lowest sequence count. Classification was done based upon the Greengenes taxonomy (adapted to mothur from [53]) with the naïve Bayesian classifier implemented in mothur (Wang algorithm). Black arrows indicate OTUs with differential abundance among the experimental conditions.

The most abundant microorganisms in the fecal microbiome were representatives of the Lachnospiraceae, Bacteroidaceae, Ruminococcacae, Enterococcaceae, Enterobacteriaceae, Verrucomicrobiaceae, Bifidobacteriaceae and Clostridiales Family XI. Incertae sedis families (Figure S3). A total of 18 different families, with documented associations with the fermentative metabolism in the gut, were investigated for their relative abundance before and after cryopreservation (Figure S6). Variable results were obtained in the different taxonomic groups (Table S2). Similar to the MOB and OLAND mixed community, most OTUs from the fecal microbiome remained present in all experimental stages and conditions, irrespective of CPA addition (Figure 5). Of all observed OTUs, 29% (representing 8.5% of total sequences) were not detected in at least one of the experimental conditions (pre-freezing, post-freezing with or without CPA). This percentage excludes the OTU presence in the fecal inoculum due to the drastic change of the community upon first cultivation. Addition of CPA during cryopreservation of the fecal biomass was necessary to maintain comparable community dissimilarities to the reference activity test (Figure 6D), but differences in community dissimilarity between the conditions with and without CPA were not significant (p = 0.24).

Overall, constrained correspondence analysis (CCA) showed that the global community structure of samples with an added CPA at t_3_ is moderately closer to the samples after the first incubation (t_1_). No significant correlations with VFA production were found with marginal effects permutation tests however with sequential effects a significant correlation was found with an increased recovery of acetic acid production (Figure S10).

## Discussion

The aim of this research was to evaluate a cryopreservation design allowing availability of a functionally and structurally reproducible inoculum for scientific and technological applications. A satisfactory recovery of specific activity of the three bacterial mixed communities studied was achieved. A critical evaluation of the community structure and differences in relative abundances or membership of the community constituents confirmed community stability, thus guaranteeing functionalities in future performance in distinct set-ups.

### 1. Addition of a CPA Enhances Fast Activity Recovery of Autotroph-driven Consortia

Previous work on pure cultures of fastidious bacteria, such as AnAOB [20], AerAOB [19], NOB [21] and MOB [18] has shown that these bacteria require addition of the appropriate CPA for survival during cryopreservation [16]. This was indeed confirmed with the MOB mixed culture and the AnAOB and AerAOB (with DMSO+TT) in the OLAND mixed community. Gel-entrapped AerAOB and NOB (denitrifying sludge) were previously shown to have better preservation in the absence of a CPA [26]. However neither DMSO nor DMSO+TT were evaluated for the gel-entrapped sludge. Also, in the current study, OLAND biomass was not gel entrapped but part of a RBC biofilm. In contrast to the findings on gel entrapped AerAOB, addition of a CPA (DMSO+TT) enabled AerAOB recovery comparable to the initial activity in this experiment. The increase in activity recovery on DMSO+TT and the reduced recovery on DMSO alone corroborate with earlier findings for *Nitrosomonas* spp. [19] for preservation at −80°C. No pre-preservation growth was executed with TT medium, which is known to enhance activity recovery of certain *Nitrosomonas* spp. [19]. Earlier findings for AnAOB showed that addition of TT along with DMSO without any pre-incubation enhanced recovery over DMSO without TT [20]. These findings aid in elucidating the overall impact of the time point of carbon addition to the preservation medium, which remain poorly understood.

The findings for NOB corroborate with earlier findings for gel entrapped NOB, since their best activity recovery was realized without CPA, as well as with more recent findings, where nearly all NOB strains resuscitated well after cryopreservation without addition of a CPA [21]. Furthermore, it has been shown that the optimal DMSO concentration for cryopreservation of certain NOB is 10% (v/v) whereas in the current study only 5% DMSO was evaluated [21].

Previous research on cryopreservation of the entire OLAND consortium at −20°C showed that AnAOB activity recovery failed [22]. To our knowledge, the current work is the first report of a successful AnAOB activity recovery after cryopreservation of the OLAND mixed community, however previous reports of cryopreservation of both aggregated and single-cell highly enriched AnAOB cultures exist [20].

For the fecal biomass, the SCFA production is a result of the saccharolytic metabolism of several cross-feeding heterotrophic bacteria in the community. Because of the high community diversity, the non-fastidious nature of heterotrophic bacteria and the rich nutritional background from where this mixed culture originated, the biomass seemed to be more ‘robust’ to cryopreservation. This was clearly demonstrated by the fact that addition of a CPA did not markedly improve activity recovery. The finding that a heterotrophic microbial consortium was not aided in fast recovery by the addition of a CPA is in contrast with earlier findings [24] for methanol-fed denitrifying biomass.

### 2. Preserving Community Structure

It has been established that different preservation conditions (i.e. a different CPA) influence the success of cryopreservation with a great variability among pure cultures on a species- or even strain-level [55]. In the case of the mixed methanotrophic community, both the effects of cryopreservation on the key ecosystem drivers (the MOB) as well as the peripheral heterotrophic community [32], [56]–[59] are of interest. Concerning the autotrophic mixed community drivers, type I and type II MOB show distinct ecophysiological features [56] and have been suggested to possess different life strategies [60]. Hence, to allow a mixed methanotrophic culture to perform in a broad range of circumstances, representatives of both type I and type II MOB should be preserved during cryopreservation. Both type I and type II MOB were recovered after cryopreservation. *Methylocystaceae* (type II MOB) did not require addition of a CPA to maintain relative abundances in the mixed culture. As our analyses were DNA-based, it is possible that the detected type II MOB are part of the microbial seed bank in the reactor (as previously demonstrated for soils [61], [62]). Type II MOB are known to have more persistent resting cells than type I MOB [63]; hence, the addition of a CPA does not influence their cryopreservation. This is in agreement with the diagnostic microarray results where the least changes in MOB diversity occurred when no CPA was added (the micro-array was only run on the NMS samples) and where *Methylocystis* sp. (strain M or related) was reduced in relative abundance when no CPA was added (Figure S7).

It has been shown that methanotrophs support heterotrophic bacteria by supplying the carbon-source for methanotrophic mixed culture. Little is known about the interactions between the methanotrophs and heterotrophs [57]. Nonetheless, these interactions are very specific [32], [57], [64], [65] and allow for adaptability to a broad range of conditions [32]. Because of the importance of these interactions [30], [66], [67], investigation of the total community structure before and after cryopreservation was performed within the scope of this study. Although, a differential impact was seen on 21% of the MOB community, this was not linked to phylogeny, even at the genus level. For instance, most OTUs classified as *Methylotenera* occurred in every experimental condition, while others were enriched after cryopreservation, and even others required addition of a CPA for cryopreservation on dNMS. Furthermore, OTUs belonging to *Devosia*, *Methylobacillus*, *Rubrivivax* required a CPA for cryopreservation on NMS. All manipulations were performed at 4°C to avoid DMSO cytotoxicity and, while some OTUs did not survive when DMSO alone was used as a CPA, no single taxonomic group was found to be more sensitive than others.

The autotrophic drivers in the OLAND community consist of relatively small part of the total community, accounting for about 43–61% of the total bacteria in a RBC biofilm and 58–74% in a granule [68], [69]. For an OLAND RBC biofilm, AerAOB, AnAOB and NOB were present at 10–28%, 33% and <5% of total cells, respectively, as determined by FISH [68]. In the current study, 6% of the total community could be classified as AerAOB, 10% as AnAOB and 0.1% as NOB. The comparatively low percentage of AnAOB might be due to underrepresentation of the *Planctomycetes* phylum in the current 16S rRNA gene sequence databases [70] or, until recently, the lack of a proper PCR protocol for the phylum [71]. Interestingly, even though NOB have a higher relative abundance with DMSO+TT after cryopreservation, their activity recovery was the lowest. This might result from a competition for nitrite with AnAOB that have the best activity recovery when DMSO+TT was used as a CPA. The increased activity recovery of AnAOB could result from the effect of DMSO on the phospholipid bilayer [72] of intracytoplasmatic membranes which contain the key enzymes for ammonium oxidation [19]. Besides the autotrophic functional community members, filamentous bacteria from the phylum *Bacteriodetes* and bacteria belonging to the phylum *Actinobacteria* were described [69] in the OLAND biomass. However, not much is known about the role of the peripheral heterotrophic community in the OLAND community. The current OLAND community shows presence of both *Actinobacteria* and *Bacteroidetes*. Only one genus required CPA addition for every representative to be cryopreserved: *Leptonema*. Some, but not all, unclassified *Rhizobiales* required the addition of TT to DMSO whereas this addition was a prerequisite for the recovery of the sole representative of the *Bdellovibrionales* order. All representatives of *Geosporobacter thermotalea, Thauera, Anaerovorax, Methylomonas, Peptinophilus, Bacteroides* and *Desulfovibrio* were enriched after cryopreservation. The only representative of the *Veillonella* genus occurs only after cryopreservation with a CPA. Apart from the peripheral heterotrophic community, peripheral autotrophs such as MOB were also detected in OLAND biomass [45], and could mitigate methane emissions from the OLAND WWTP. Type I MOB were detected in all conditions after cryopreservation up to 0.05% of relative abundance in the conditions where DMSO and DMSO+TT were added.

In the fecal community, many different taxonomic families were implicated in the SCFA production. Because of the high diversity and number of representatives in most taxonomic levels, no clear influence of cryopreservation on taxonomic group representation in the fecal microbiome was discerned. The only existing study on cryopreservation of vertebrate fecal biomass shows that addition of a CPA aided in recovery of the growth of bacterial cells [73].

Some OTUs (8% (MOB, OLAND) to 15% (Fecal community)) were not detected at t_0_ but do occur at t_1_ or t_3_. The most probable explanation is a very low sequence count of these OTUs in the initial inoculum which might have been either processed out in OTU binning or “rarefied out” when subsampling to lowest sample sequence count.

In contrast to the investigation of individual (taxonomic) community changes, the assessment of overall community structure is a more robust approach to uncover community structure. This approach has a greater ecological and methodological relevance as it aims at quantifying the global community changes rather than relying upon classification and taxonomy. It is clear that the Jaccard dissimilarity was less when a CPA was added during cryopreservation for each of the evaluated microbial cultures. Constrained canonical correspondence analysis integrates both functionality and community structure data. This analysis supports the conclusions from the comparison of Jaccard dissimilarities.

## Conclusion and Perspectives

A cryopreservation protocol for mixed microbial cultures was evaluated over three months with three different bacterial mixed cultures. The use of DMSO + trehalose and tryptic soy broth as a CPA consistently gave the best success rate although the cryopreservative was not necessary to obtain adequate cryopreservation of fecal material. The functionality recovery in a three-month cryopreservation experiment was previously shown to be similar in longer duration experiments (6–12 months [18]).

From an ecological point of view, even with CPA addition, not all OTUs were preserved. However, no significant differences in overall community structure were found. Although a perfect preservation of community structure was not obtained, one might question the importance of a single OTU in the community structure.

From a biotechnological point of view, CPA addition was necessary for fast and reproducible activity recovery. Only with the fecal material, optimization of the method is necessary. Overall a reproducible storage method was found where addition of DMSO+TT as a CPA outperforms the limited state-of-the art preservation techniques for mixed microbial cultures. As adequate activity recovery can be obtained without introduction of an extended lag phase, undoubtedly this methodology will boost the use of mixed cultures in biotechnological applications.

## Supporting Information

Figure S1 **Relative abundances of taxa in the MOB samples.** The top-8 taxa are displayed. The RDP classifier, reference set and taxonomy were used. The deepest possible classification is given up to the family level. The dataset was rarefied to the sample with the lowest sequence count after removal of the anomalous samples (data not shown). Relative abundances were calculated on a sample-wise basis after summing the sequence counts of the OTUs that could be classified on the family level.
(TIF)

Figure S2 **Relative abundances of taxa in the OLAND samples.** The top-7 taxa are displayed. The RDP classifier, reference set and taxonomy were used. The deepest possible classification is given up to the family level. The dataset was rarefied to the sample with the lowest sequence count. Relative abundances were calculated on a sample-wise basis.
(TIF)

Figure S3 **Relative abundances of taxa in the fecal biomass samples.** The top-8 taxa are displayed. The RDP classifier, reference set and taxonomy were used. The deepest possible classification is given up to the family level. The dataset was rarefied to the sample with the lowest sequence count. Relative abundances were calculated on a sample-wise basis.
(TIF)

Figure S4 **Relative abundance of methanotrophic families within the rarefied dataset.** Bar heights represent means of duplicate (Inoculum and all post samples) or triplicate (pre samples) reactors. Error bars represent the respective standard deviations. Bars with equal letters are not significantly different at the 95% significance level.
(TIF)

Figure S5 **Relative abundance of relevant families for the OLAND process within the rarefied dataset.**
(TIF)

Figure S6 **Relative abundance of relevant families for the SCFA production within the fecal community within the rarefied dataset.** Classification was done with the SILVA v111 database and the SINA aligner. Axes are not constant. The error bars represent the standard deviation of biological duplicate incubations. The fecal inoculum is displayed, as a reference (n = 1).
(TIFF)

Figure S7 **MOB diversity microarray results.**
(PDF)

Figure S8 **Rarefaction curves.** Colors represent sample type as used in the phylogenetic trees. A) MOB rarefaction. Samples 1 & 2: inoculum (t_0_); 3 & 4 NMS t_1_; 5–7 dNMS t_1_; 8 & 9: No CPA NMS; 10 & 11 No CPA dNMS; 12 & 13: DMSO NMS; 14 & 15 DMSO dNMS; 16 & 17 DMSO+TT NMS; 18 & 19 DMSO+TT dNMS. The rarefied dataset was subsampled at 16591 sequences per sample. B) for the OLAND biomass. Pooled samples: 1: t_0_, 2: No CPA, 3: DNMSO, 4: DMSO+TT. The rarefied dataset was subsampled at 17647 sequences per sample. C) for the fecal microbiome. 1 & 2: t_1_; 3 & 4: No CPA; 5 & 6: DMSO; 7 & 8: DMSO+TT; 9: fecal inoculum (t_0_). The rarefied dataset was subsampled at 12440 sequences per sample.
(TIF)

Figure S9 **Partial Constrained Correspondence Analysis ((p)CCA) ordination graph for the MOB community.** The analysis was constrained (26% of total inertia) on the MOR (p = 0.01) and conditioned (2% of total inertia) on media (NMS/dNMS). The red arrow represents increasing MOR. Shapes with a dark color represent samples incubated with NMS whilst shapes with a light color represent samples incubated on dNMS. The green triangles correspond to the samples cryopreserved without CPA (t_3_). Orange/gold circles represent samples cryopreserved with DMSO+TT (t_3_). Purple diamonds represent samples cryopreserved with only DMSO as a CPA (t_3_). Blue circles represent samples after the reference activity test (t_1_) and red squares represent the original inoculum (t_0_). Clusters of samples are highlighted. The distance between individual samples was calculated based upon the abundance-based Jaccard index.
(TIF)

Figure S10 **Constrained Correspondence Analysis (CCA) ordination graph for the fecal community.** The fecal inoculum was removed from the analysis. The analysis was constrained (81% of total inertia) on the concentrations of acetic acid (p = 0.02), propionic acid (p = 0.37) and butyric acid (p = 0.76). The red arrows represent increasing SCFA concentrations. The green triangles correspond to the samples cryopreserved without CPA (t_3_). Orange/gold circles represent samples cryopreserved with DMSO+TT (t_3_). Purple diamonds represent samples cryopreserved with only DMSO as a CPA (t_3_). Blue circles represent samples after the reference activity test (t_1_) and The distance between individual samples was calculated based upon the abundance-based Jaccard index.
(TIF)

Table S1 **Composition of the trace element solution for NMS and dNMS.**
(DOCX)

Table S2 **Results of cryopreservation of 18 bacterial families from the fecal biomass implicated in SCFA production.**
(DOCX)

Dataset S1 **Sequences and their counts in the MOB samples.**
(XLSX)

Dataset S2 **Sequences and their counts in the OLAND samples.**
(XLSX)

Dataset S3 **Sequences and their counts in the Fecal samples.**
(XLSX)
