# Supplementary figures and images for: Optimized Cryopreservation of Mixed Microbial Communities for Conserved Functionality and Diversity
Source: PLoS One. 2014 Jun 17;9(6):e99517. doi: 10.1371/journal.pone.0099517 (PMC4061060; doi:10.1371/journal.pone.0099517)

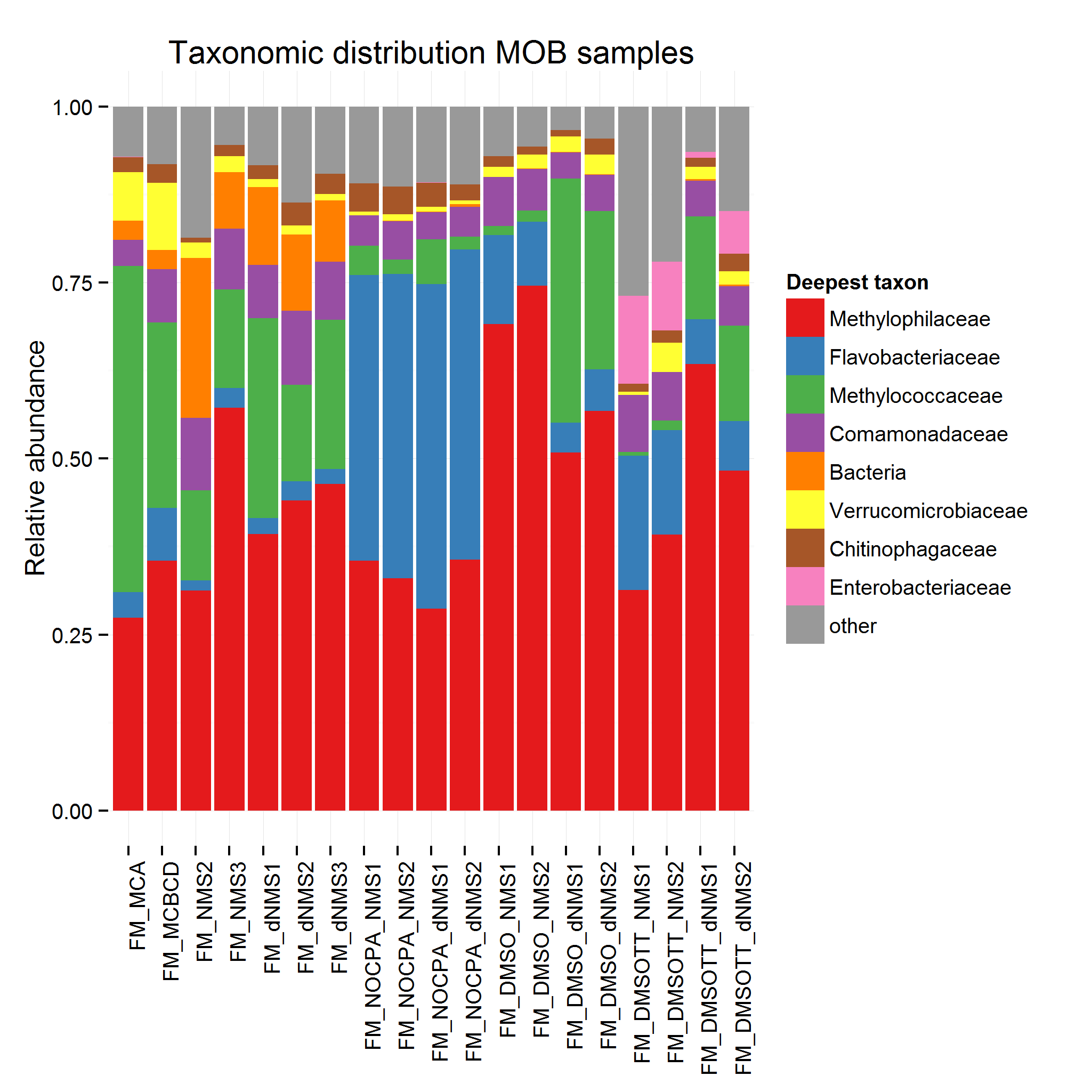

Supplement: Figure S1 — Relative abundances of taxa in the MOB samples. The top-8 taxa are displayed. The RDP classifier, reference set and taxonomy were used. The deepest possible classification is given up to the family level. The dataset was rarefied to the sample with the lowest sequence count after removal of the anomalous samples (data not shown). Relative abundances were calculated on a sample-wise basis after summing the sequence counts of the OTUs that could be classified on the family level. (TIF) [file pone.0099517.s001.tif]

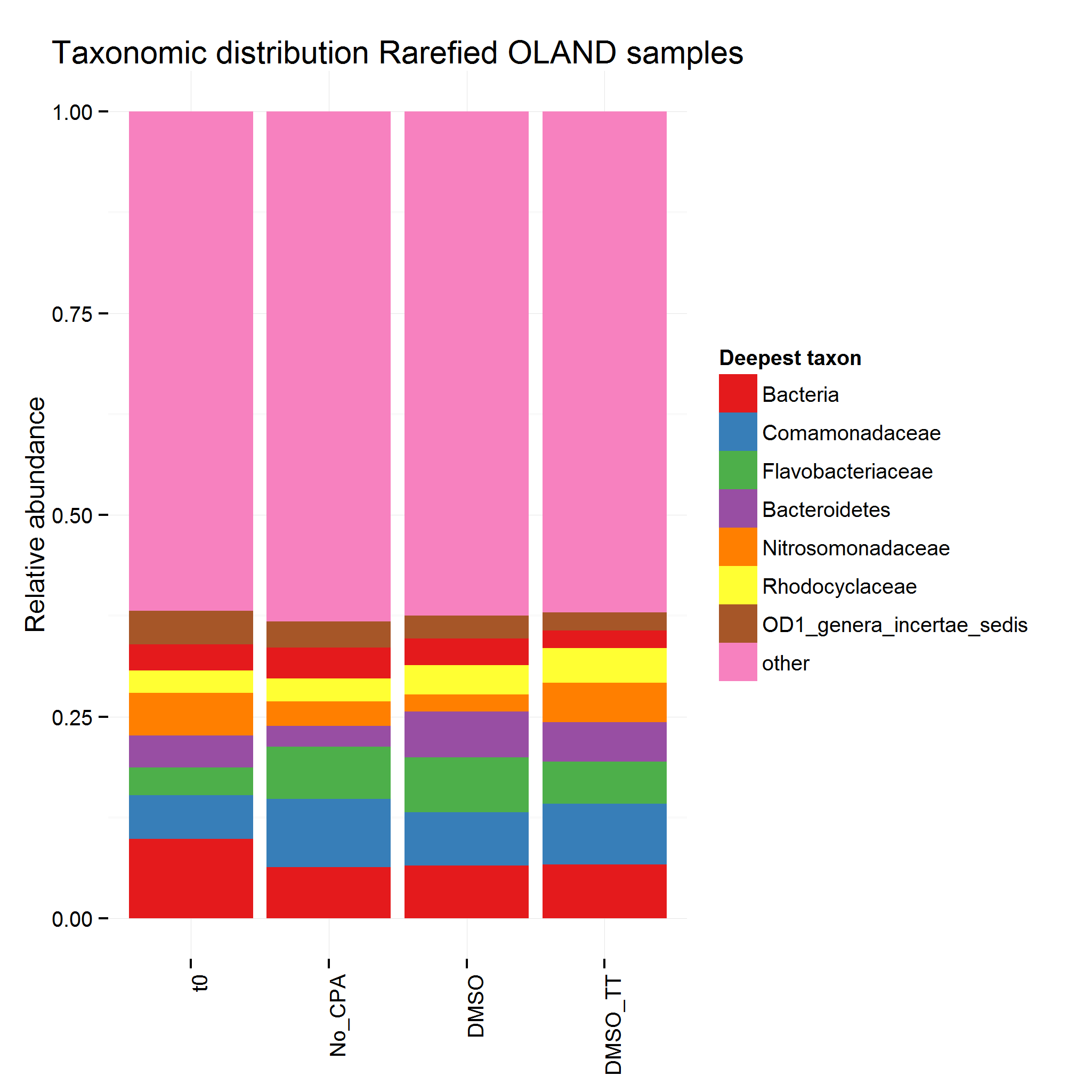

Supplement: Figure S2 — Relative abundances of taxa in the OLAND samples. The top-7 taxa are displayed. The RDP classifier, reference set and taxonomy were used. The deepest possible classification is given up to the family level. The dataset was rarefied to the sample with the lowest sequence count. Relative abundances were calculated on a sample-wise basis. (TIF) [file pone.0099517.s002.tif]

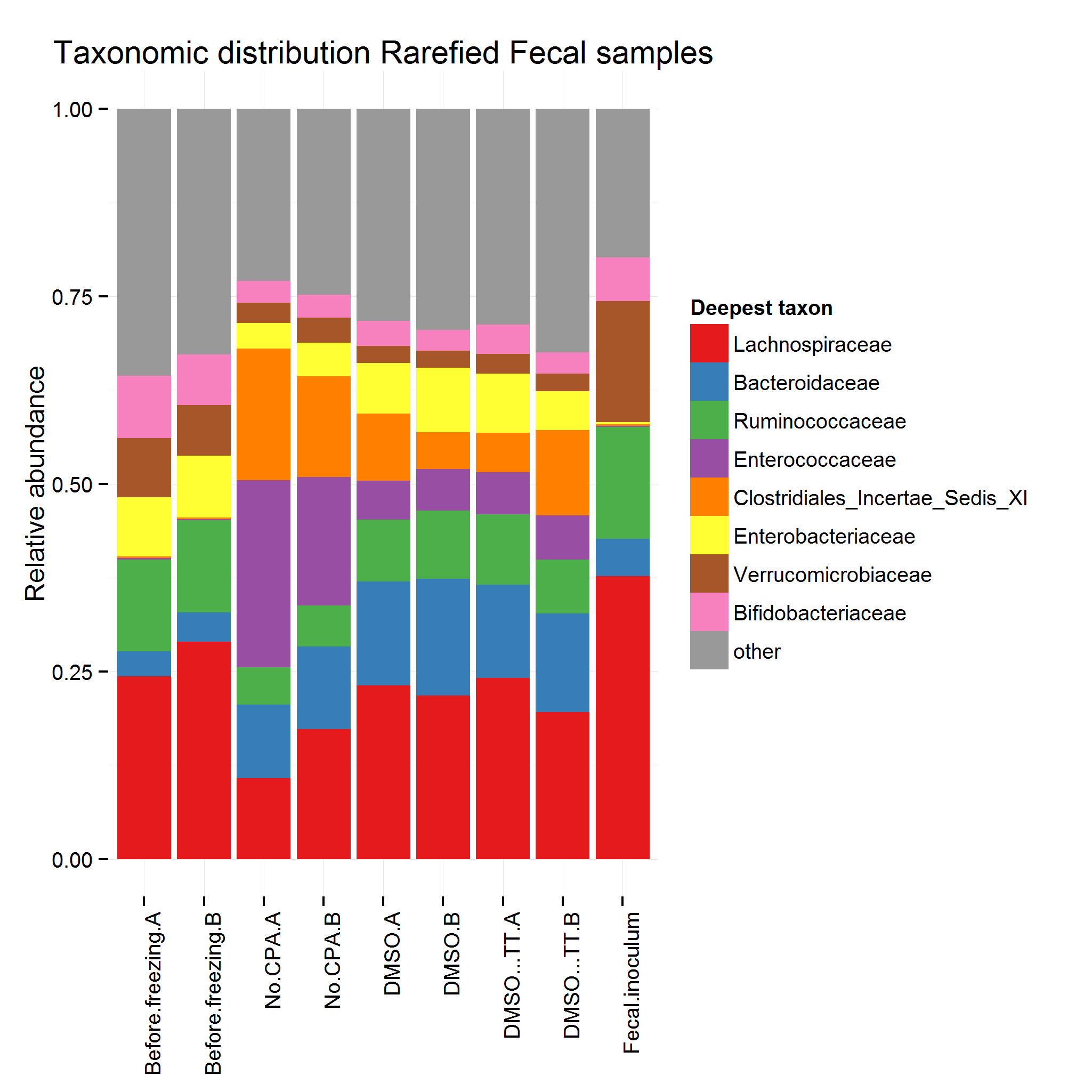

Supplement: Figure S3 — Relative abundances of taxa in the fecal biomass samples. The top-8 taxa are displayed. The RDP classifier, reference set and taxonomy were used. The deepest possible classification is given up to the family level. The dataset was rarefied to the sample with the lowest sequence count. Relative abundances were calculated on a sample-wise basis. (TIF) [file pone.0099517.s003.tif]

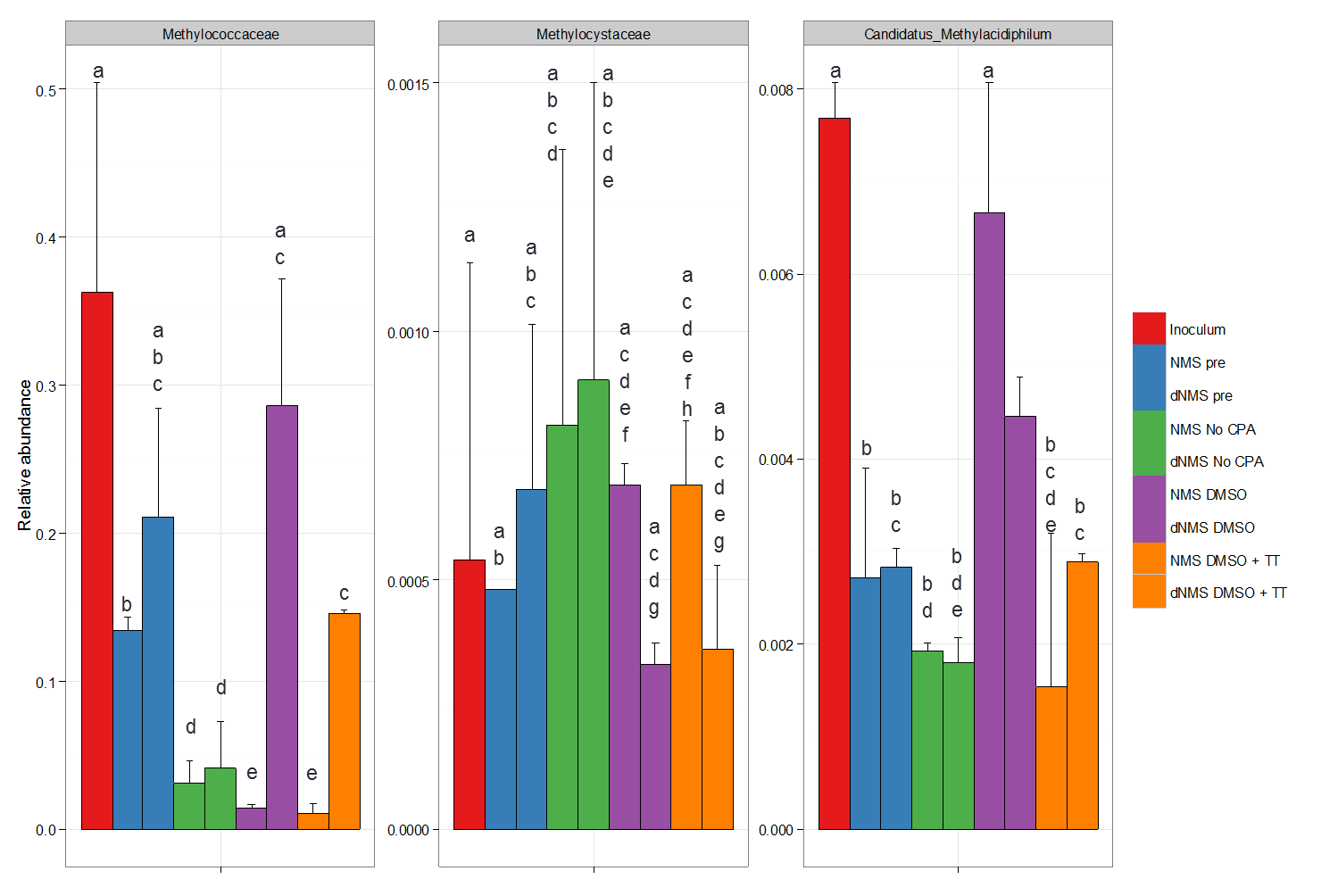

Supplement: Figure S4 — Relative abundance of methanotrophic families within the rarefied dataset. Bar heights represent means of duplicate (Inoculum and all post samples) or triplicate (pre samples) reactors. Error bars represent the respective standard deviations. Bars with equal letters are not significantly different at the 95% significance level. (TIF) [file pone.0099517.s004.tif]

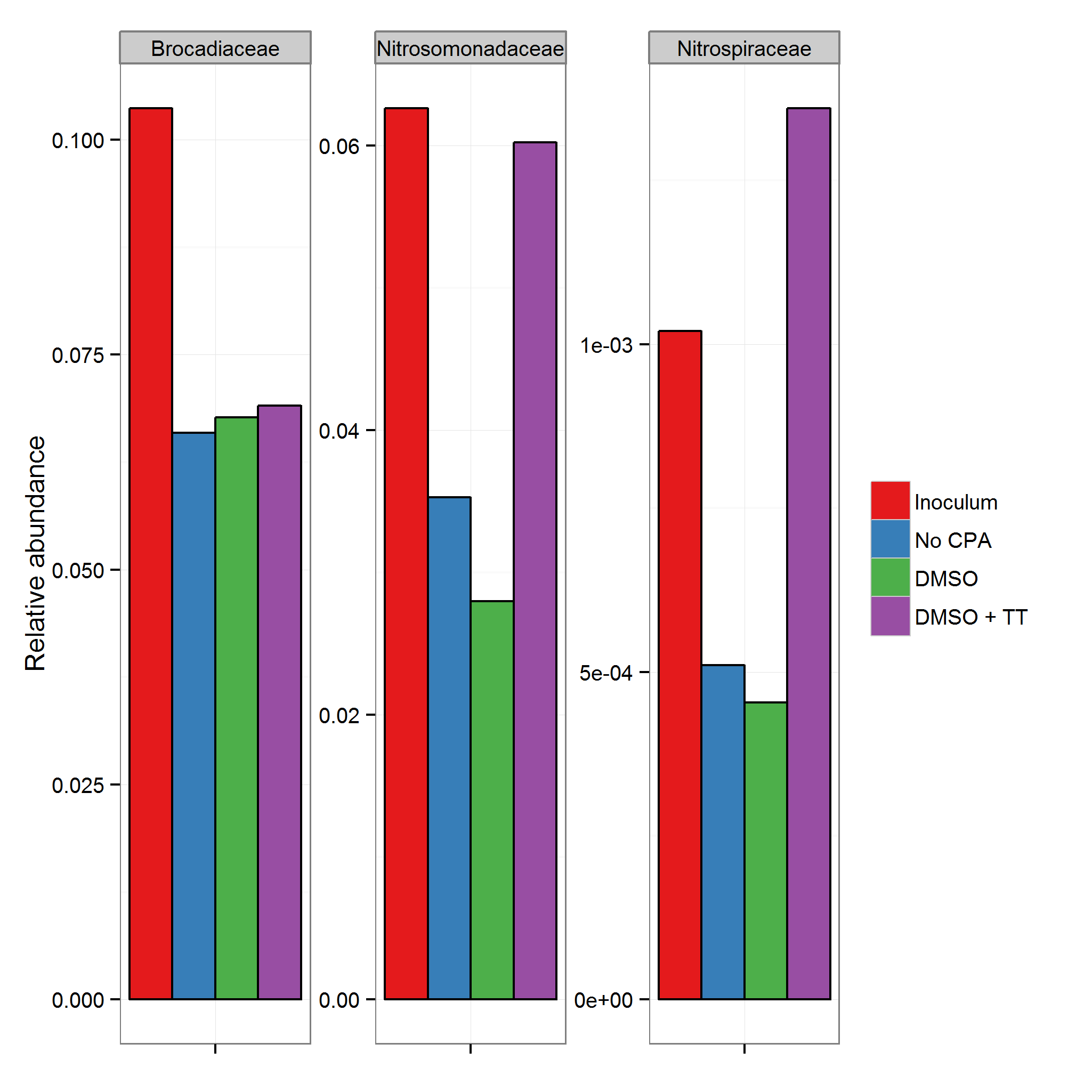

Supplement: Figure S5 — Relative abundance of relevant families for the OLAND process within the rarefied dataset. (TIF) [file pone.0099517.s005.tif]

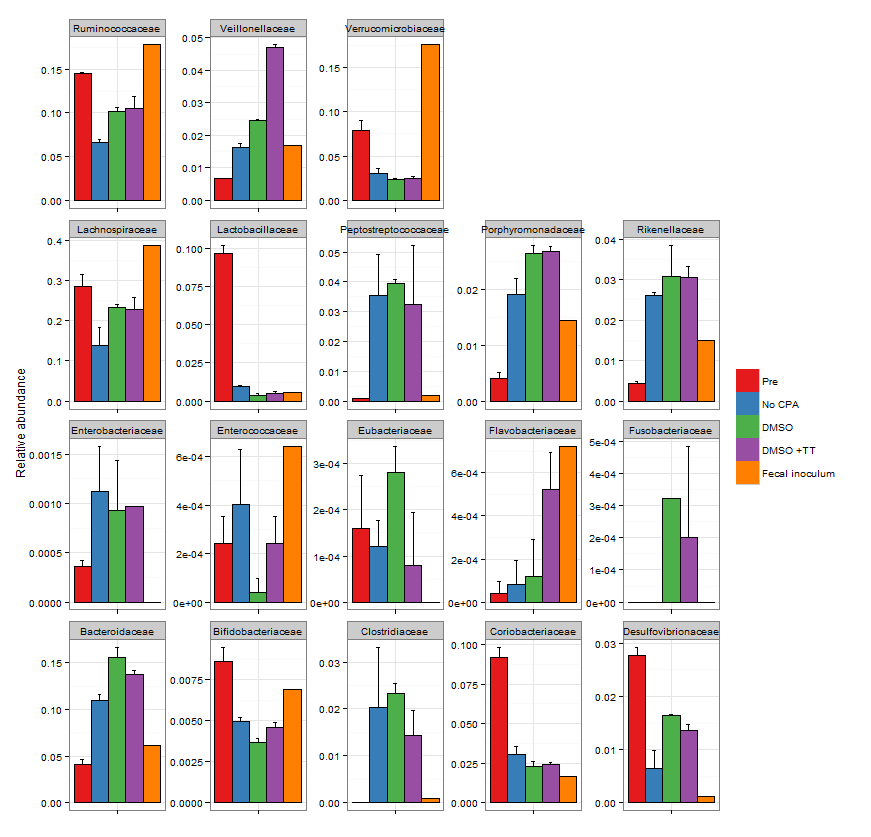

Supplement: Figure S6 — Relative abundance of relevant families for the SCFA production within the fecal community within the rarefied dataset. Classification was done with the SILVA v111 database and the SINA aligner. Axes are not constant. The error bars represent the standard deviation of biological duplicate incubations. The fecal inoculum is displayed, as a reference (n = 1). (TIFF) [file pone.0099517.s006.tiff]

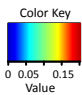

Probes

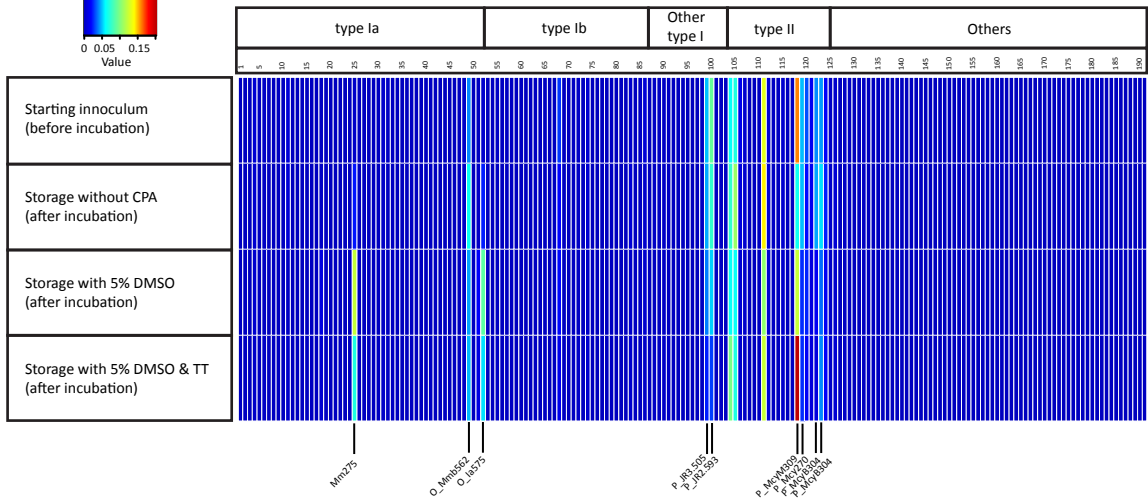

Supplement: Figure S7 — MOB diversity microarray results. (PDF) [file pone.0099517.s007.pdf]

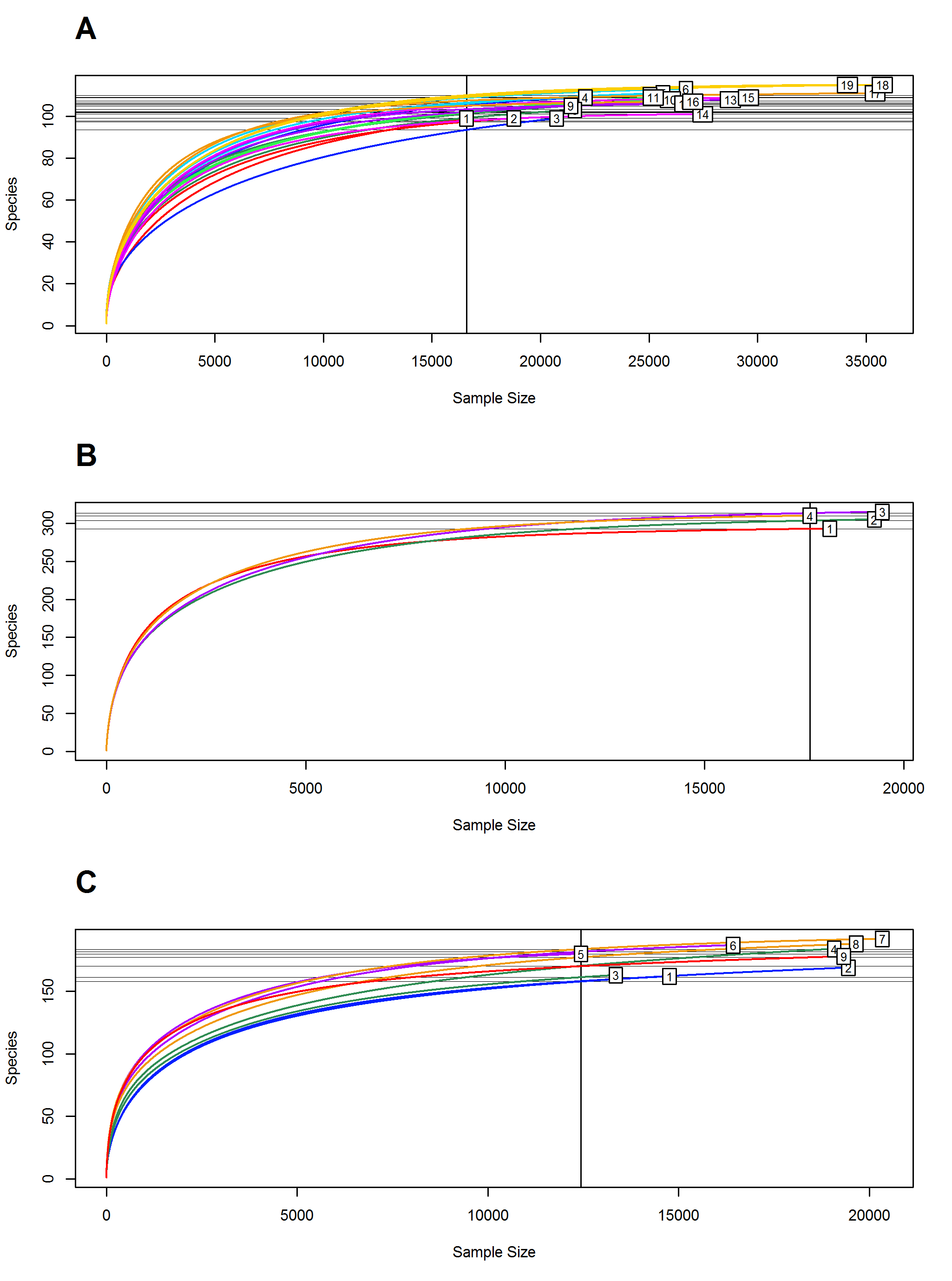

Supplement: Figure S8 — Rarefaction curves. Colors represent sample type as used in the phylogenetic trees. A) MOB rarefaction. Samples 1 & 2: inoculum (t0); 3 & 4 NMS t1; 5–7 dNMS t1; 8 & 9: No CPA NMS; 10 & 11 No CPA dNMS; 12 & 13: DMSO NMS; 14 & 15 DMSO dNMS; 16 & 17 DMSO+TT NMS; 18 & 19 DMSO+TT dNMS. The rarefied dataset was subsampled at 16591 sequences per sample. B) for the OLAND biomass. Pooled samples: 1: t0, 2: No CPA, 3: DNMSO, 4: DMSO+TT. The rarefied dataset was subsampled at 17647 sequences per sample. C) for the fecal microbiome. 1 & 2: t1; 3 & 4: No CPA; 5 & 6: DMSO; 7 & 8: DMSO+TT; 9: fecal inoculum (t0). The rarefied dataset was subsampled at 12440 sequences per sample. (TIF) [file pone.0099517.s008.tif]

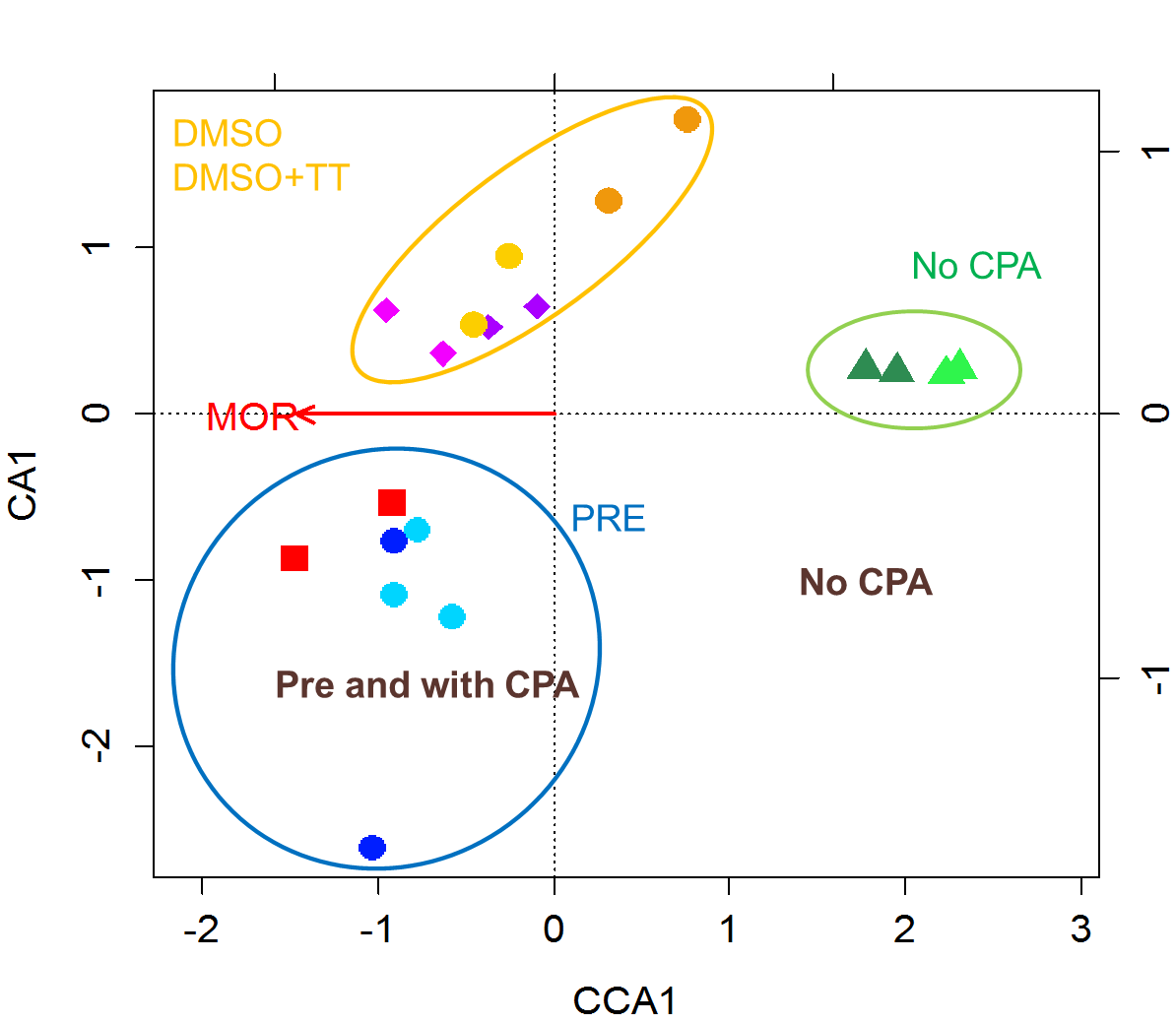

Supplement: Figure S9 — Partial Constrained Correspondence Analysis ((p)CCA) ordination graph for the MOB community. The analysis was constrained (26% of total inertia) on the MOR (p = 0.01) and conditioned (2% of total inertia) on media (NMS/dNMS). The red arrow represents increasing MOR. Shapes with a dark color represent samples incubated with NMS whilst shapes with a light color represent samples incubated on dNMS. The green triangles correspond to the samples cryopreserved without CPA (t3). Orange/gold circles represent samples cryopreserved with DMSO+TT (t3). Purple diamonds represent samples cryopreserved with only DMSO as a CPA (t3). Blue circles represent samples after the reference activity test (t1) and red squares represent the original inoculum (t0). Clusters of samples are highlighted. The distance between individual samples was calculated based upon the abundance-based Jaccard index. (TIF) [file pone.0099517.s009.tif]

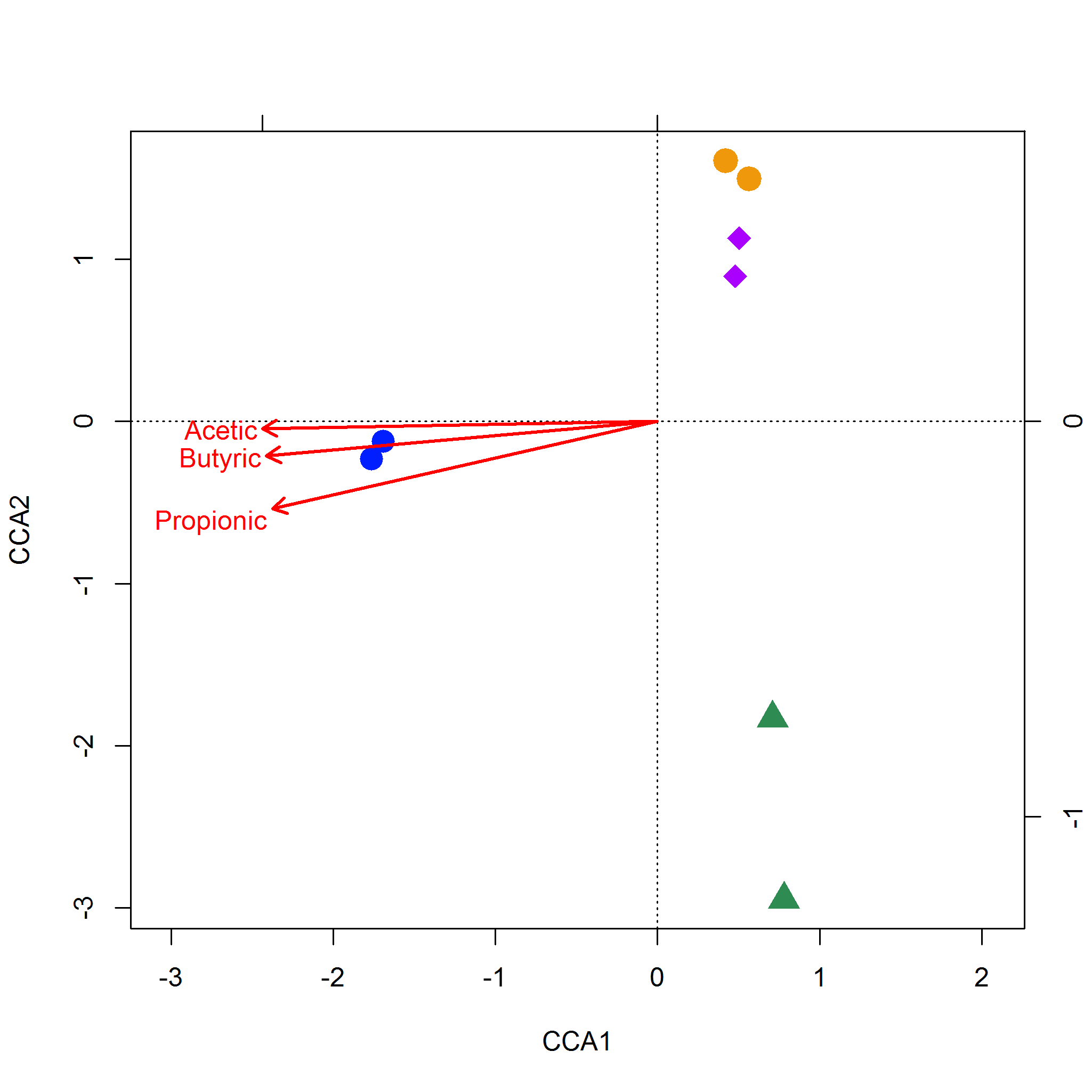

Supplement: Figure S10 — Constrained Correspondence Analysis (CCA) ordination graph for the fecal community. The fecal inoculum was removed from the analysis. The analysis was constrained (81% of total inertia) on the concentrations of acetic acid (p = 0.02), propionic acid (p = 0.37) and butyric acid (p = 0.76). The red arrows represent increasing SCFA concentrations. The green triangles correspond to the samples cryopreserved without CPA (t3). Orange/gold circles represent samples cryopreserved with DMSO+TT (t3). Purple diamonds represent samples cryopreserved with only DMSO as a CPA (t3). Blue circles represent samples after the reference activity test (t1) and The distance between individual samples was calculated based upon the abundance-based Jaccard index. (TIF) [file pone.0099517.s010.tif]
