# Supplementary material for: Optimized Cryopreservation of Mixed Microbial Communities for Conserved Functionality and Diversity
Source: PLoS One. 2014 Jun 17;9(6):e99517. doi: 10.1371/journal.pone.0099517 (PMC4061060; doi:10.1371/journal.pone.0099517)
Supplement: Table S1 — Composition of the trace element solution for NMS and dNMS. (DOCX) [file pone.0099517.s011.docx]

| Na_2_EDTA.2H_2_O | 0.5 g L^-1^ | MnCl_2_.4H_2_O | 0.003 g L^-1^ |
| --- | --- | --- | --- |
| FeSO_4_.7H_2_O | 0.2 g L^-1^ | NaMoO_4_.2H_2_O | 0.003 g L^-1^ |
| H_3_BO_3_ | 0.03 g L^-1^ | NiCl_2_.6H_2_O | 0.002 g L^-1^ |
| CoCl_2_.6H_2_O | 0.02 g L^-1^ | CuSO_4_.5H_2_O | 2.5 g L^-1^ |
| ZnSO_4_.7H_2_O | 0.01g L^-1^ |  |  |
