# Supplementary material for: Optimized Cryopreservation of Mixed Microbial Communities for Conserved Functionality and Diversity
Source: PLoS One. 2014 Jun 17;9(6):e99517. doi: 10.1371/journal.pone.0099517 (PMC4061060; doi:10.1371/journal.pone.0099517)
Supplement: Table S2 — Results of cryopreservation of 18 bacterial families from the fecal biomass implicated in SCFA production. (DOCX) [file pone.0099517.s012.docx]

| Family | Effect |
| --- | --- |
| *Ruminococcaceae* | Better cryopreservation when CPA was added, however overall reduced abundance. No difference between DMSO and DMSO+TT |
| *Veillonellaceae* | Enrichment after cryopreservation mainly with DMSO+TT |
| *Verrucomicrobiaceae* | Reduced abundance after cryopreservation regardless of CPA addition |
| *Lachnospiraceae* | Better cryopreservation when CPA was added, no difference between DMSO and DMSO+TT |
| *Lactobacillaceae* | Reduced abundance after cryopreservation, regardless of CPA addition |
| *Peptostreptococcaceae* | Enrichment after cryopreservation, regardless of CPA addition |
| *Porphyromonadaceae* | Enrichment after cryopreservation, mainly when a CPA was added |
| *Rikenellaceae* | Enrichment after cryopreservation, regardless of CPA addition |
| *Enterobacteriaceae* | Not affected by cryopreservation, regardless of CPA addition |
| *Enterococcaceae* | Not affected cryopreservation but only without CPA addition or with addition of DMSO+TT as a CPA |
| *Eubacteriaceae* | Not affected by cryopreservation, enrichment with DMSO |
| *Flavobacteriaceae* | Not affected by cryopreservation, enrichment with DMSO+TT |
| *Fusobacteriaceae* | Enrichment with CPA addition |
| *Bacteroidaceae* | Enrichment after cryopreservation, and more strongly with CPA addition |
| *Bificobacteriaceae* | Reduced abundance regardless of CPA addition. Better without CPA |
| *Clostridiaceae* | Enrichment after cryopreservation |
| *Coriobacteriaceae* | Reduced abundance after cryopreservation, regardless of CPA addition |
| *Desulfovibrionaceae* | Reduced abundance after cryopreservation, but better performance of DMSO and, to a lesser extent, DMSO+TT |
